# Methodological standards, quality of reporting and regulatory compliance in animal research on amyotrophic lateral sclerosis: a systematic review

**DOI:** 10.1136/bmjos-2018-000016

**Published:** 2019-08-01

**Authors:** Joana G Fernandes, Nuno H Franco, Andrew J Grierson, Jan Hultgren, Andrew J W Furley, I Anna S Olsson

**Affiliations:** 1 Instituto de Investigação e Inovação em Saúde, Universidade do Porto, Porto, Portugal; 2 IBMC-Instituto de Biologia Molecular e Celular, Universidade do Porto, Porto, Portugal; 3 Department of Neuroscience, Sheffield Institute for Translational Neuroscience, University of Sheffield, Sheffield, UK; 4 Bateson Centre, University of Sheffield, Sheffield, UK; 5 Department of Animal Environment and Health, Swedish University of Agricultural Sciences, Skara, Sweden; 6 Department of Biomedical Science, University of Sheffield, Western Bank, Sheffield, UK

**Keywords:** amyotrophic lateral sclerosis, ALS, guidelines, methodology, reporting, quality, compliance, animal welfare, reproducibility

## Abstract

**Objectives:**

The amyotrophic lateral sclerosis (ALS) research community was one of the first to adopt methodology guidelines to improve preclinical research reproducibility. We here present the results of a systematic review to investigate how the standards in this field changed over the 10-year period during which the guidelines were first published (2007) and updated (2010).

**Methods:**

We searched for papers reporting ALS research on SOD1 (superoxide dismutase 1) mice published between 2005 and 2015 on the ISI Web of Science database, resulting in a sample of 569 papers to review, after triage. Two scores—one for methodological quality, one for regulatory compliance—were built from weighted sums of separate sets of items, and subjected to multivariable regression analysis, to assess how these related to publication year, type of study, country of origin and journal.

**Results:**

Reporting standards improved over time. Of papers published after the first ALS guidelines were made public, fewer than 9% referred specifically to these. Of key research parameters, only three (genetic background, number of transgenes and group size) were reported in >50% of the papers. Information on housing conditions, randomisation and blinding was absent in over two-thirds of the papers. Group size was among the best reported parameters, but the majority reported using fewer than the recommended sample size and only two studies clearly justified group size.

**Conclusions:**

General methodological standards improved gradually over a period of 8–10 years, but remained generally comparable with related fields with no specific guidelines, except with regard to severity. Only 11% of ALS studies were classified in the highest severity level (animals allowed to reach death or moribund stages), substantially below the proportion in studies of comparable neurodegenerative diseases such as Huntington’s. The existence of field-specific guidelines, although a welcome indication of concern, seems insufficient to ensure adherence to high methodological standards. Other mechanisms may be required to improve methodological and welfare standards.

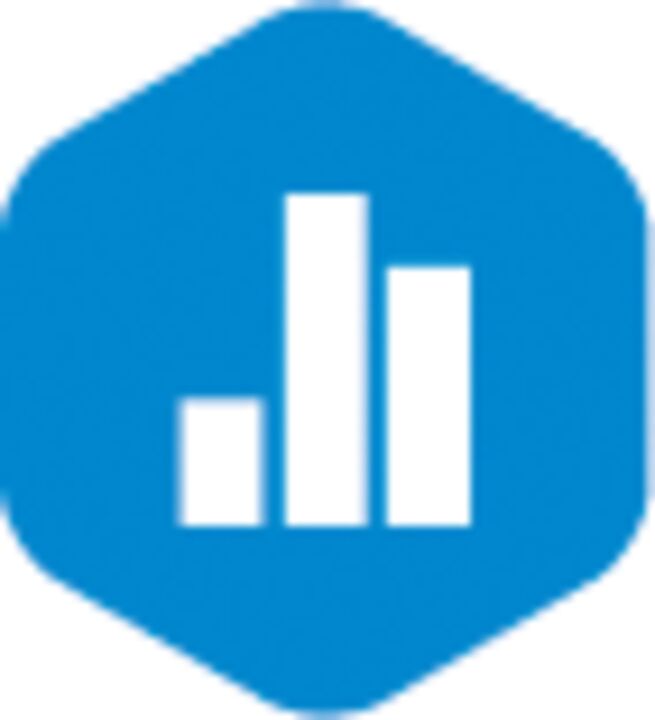

**Strengths and limitations of this study**

- The approach for this systematic review is unique in covering methodological quality, regulatory compliance and severity or animal welfare.
- We built two comprehensive scores (for methodological standards and for regulatory compliance) which were subjected to multivariable regression analysis to investigate how these scores were related to publication year, type of study, country of origin and journal, simultaneously accounting for all these factors.
- Our large sample (N=569 papers) included half the total population of published papers between 2005 and 2015.
- While more models of amyotrophic lateral sclerosis are now available, only studies using the SOD1 (superoxide dismutase 1) mouse were included.
- The protocol was defined prior to data collection but was not registered prior to the study.
- Information retrieval and assessment were not blinded.

## Introduction

Amyotrophic lateral sclerosis (ALS) is a rapidly progressing neurodegenerative disease typically resulting in death 2–5 years after the onset of symptoms. There is no known cure, and the most widely used treatment—riluzole—extends survival by just 2 months.^1^ ALS research using animal models focuses primarily on two main interconnected goals: understanding the underlying mechanisms involved in motor neuron death in the brain and spinal cord, and development and testing of potential drug therapies.^2^ This research relies substantially on genetically modified animals, particularly transgenic mice expressing mutant forms of the human superoxide dismutase 1 (SOD1) gene, which manifest several important characteristics of the human disease.^3 4^

While the use of animal models is relevant for advancing knowledge and considered essential for testing putative treatments, it also presents ethical challenges and thus may be a reason for public concern. As a result, a common legal requirement in many countries is that animal research projects undergo an evaluation process intended to ensure that protocols are designed and carried out in compliance with the 3Rs principle: *replacement* of animal use by non-animal methods, *reduction* of animal numbers needed to achieve the scientific objectives, and *refinement* of procedures to reduce or prevent harm to animals and improve their well-being. Systematic reviews of animal use in both neuroscience^5^ and infection^6^ research indicate that self-reported regulatory compliance, including of ethical approval of protocols, has steadily increased over the last decade, but that significant progress could still be made to minimise and prevent avoidable suffering of laboratory animals. One key measure for accomplishing this is the termination of experiments during less severe stages of disease development where it is scientifically valid to do so. Endpoints based on early obtainable and scientifically sound indicators of phenotype progression can improve the ethical acceptability of animal studies and prevent the confounding influence of secondary factors; in the case of animal models of neurodegenerative diseases, starvation and dehydration arising from difficulties in eating and drinking due to progressive motor impairment can affect the phenotype and the read-out of survival studies.^7–9^ Simple refinements, such as adding mashed food and longer bottle spouts, can however help reduce the influence of such factors.^10–12^

Of related concern are reports that a number of published animal studies fail to uphold basic standards regarding experimental design—for example, random assignment of animals to treatment groups, blinding of observers—or use too few animals, often leading to irreproducible results of limited translational value.^13–18^ This also holds true for neuroscience,^19–22^ with concerns over the overall quality and reproducibility of published results being raised for several neuroscience subfields, including multiple sclerosis,^23^ stroke,^24^ spinal cord injury,^25^ Alzheimer’s,^26^ Parkinson’s,^27^ Huntington’s^12^ and ALS^28^ research. This has led major science funders, including the National Institutes of Health^29^ and Research Councils UK,^30^ to demand that future grant proposals attest to the likelihood of providing reliable results, by including details of experimental design and adequate justification of sample sizes. Reproducibility is further hindered by insufficient provision of information on methodology in published research,^31^ including failure to account for key variables such as sex, genotype, age and weight of animals, anaesthetics used, or methods of euthanasia. Omitting information also makes it impossible to evaluate the study quality, and there is evidence that papers that do not report randomisation or blinding exaggerate biological effects.^32–34^

Broadly, the public conditionally approves of animal studies on the assumption that the harm caused is offset by the benefits achieved and that scientists strive to minimise the former and optimise the latter.^35 36^ Doing so requires scientists to critically revise their methods to maximise translational relevance.^18 37^ Scientists are rightly concerned and, within the self-correcting process of science, must rely on themselves to both identify the main obstacles hindering its progress and find adequate solutions. To address the issue of methodological standards and quality of reporting of basic and applied ALS studies, the ALS research community held two meetings in 2006 and 2009, resulting in the publication of guidelines for animal studies in this field.^2 38^ These guidelines aim to improve and standardise research methodology, and encourage authors and journals to publish negative results in order to avoid publication bias. The actual impact of such guidelines on how the ALS community carries out and reports research has however not been assessed.

The present systematic review of animal studies of ALS uniquely aimed to assess, over an extended period, the attention given to relevant methodological parameters (as a proxy for the likely reliability of the study) and to examine how the principles of *refinement* and *reduction* (measures to minimise animal harm) were considered. Both proof-of-concept and preclinical studies were included in order to assess the influence of the type of study.

## Methods

### Database search

**Figure 1:**
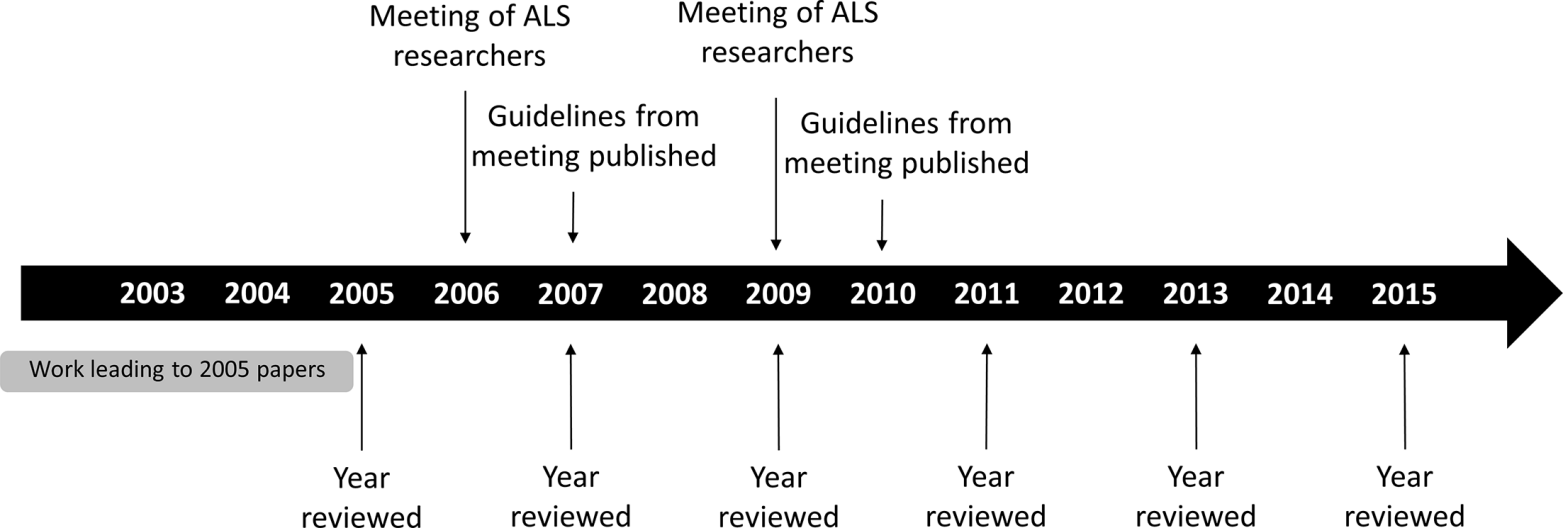
Timeline of relevant events. The bottom arrows signal the years for which papers in our sample were retrieved, and the top arrows indicate the years when workshops on best practice in ALS animal research were held, as well as when guidelines stemming from these were published. The grey bar illustrates the period of 1–4 years over which ALS animal studies reported in 2005 were likely to have been designed and carried out, an estimation that can also be applied for the other years reviewed (2007, 2009, 2011, 2013 and 2015). ALS, amyotrophic lateral sclerosis.

An advanced search was conducted on the *ISI Web of Science* database with the query *TS = ((mice OR mouse) SAME (ALS OR ‘amyotrophic lateral sclerosis’)).* The database choice followed the protocol established for our previous reviews,^5 6^ based on considerations of access, search function and wide coverage of life sciences research. Results were refined to include only original research articles written in English and published in 2005, 2007, 2009, 2011, 2013 and 2015. Years of publication were selected to include papers reporting research planned and carried out prior to and after the publication of guidelines for ALS research in 2007^38^ and 2010,^2^ resulting from two international meetings held in 2006 and 2009, respectively (figure 1).

**Figure 2:**
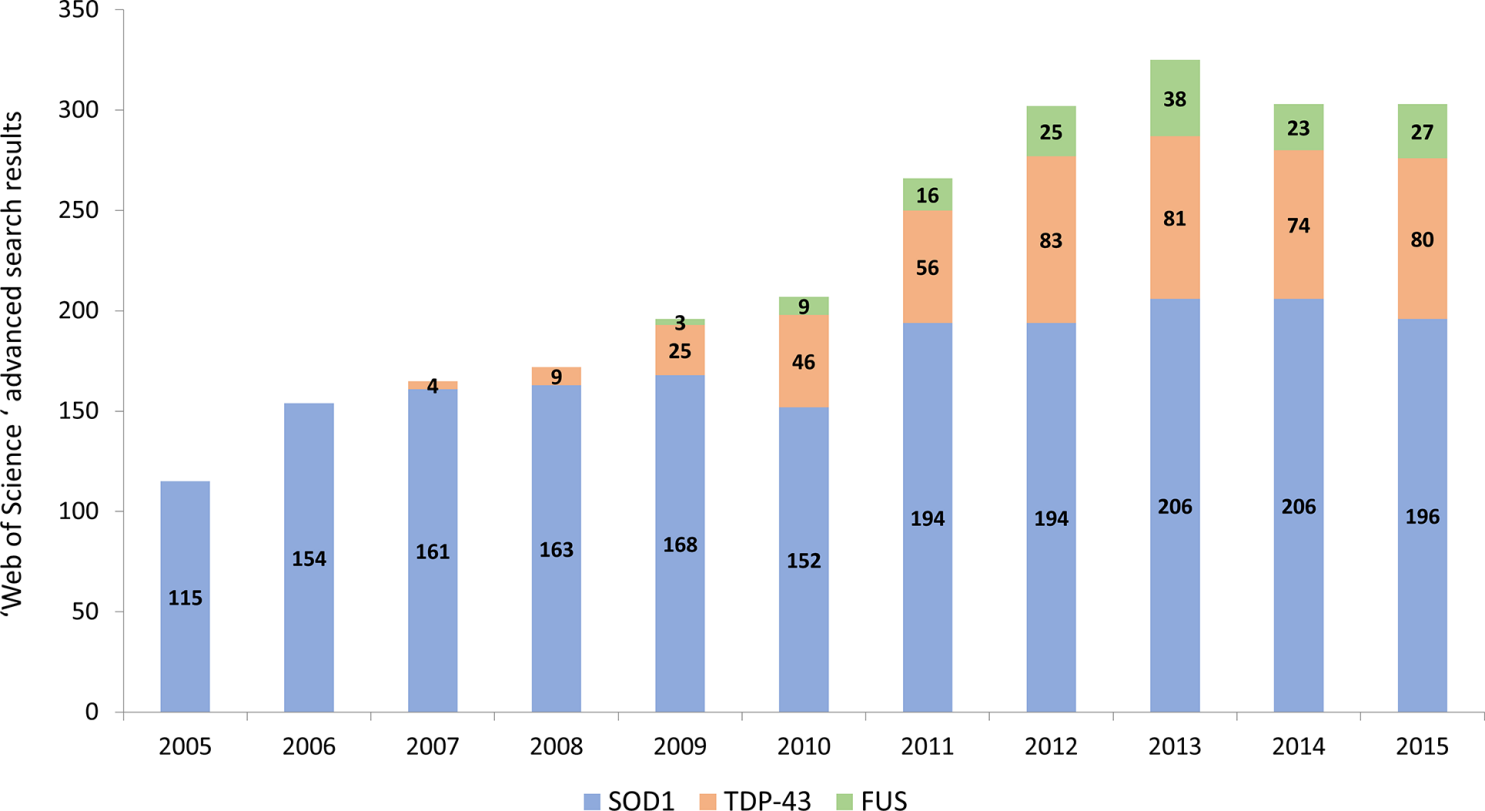
Trends in animal model chosen in ALS research, based on the number of hits from a *Clarivate Analytics Web of Science* advanced search applying the search queries: (1) TS = ((‘ALS’ OR ‘amyotrophic lateral sclerosis’) AND ‘SOD1’ AND (‘mouse’ OR ‘mice’)); (2) TS = ((‘ALS’ OR ‘amyotrophic lateral sclerosis’) AND ‘TDP-43’ AND (‘mouse’ OR ‘mice’)); and (3) TS = ((‘ALS’ OR ‘amyotrophic lateral sclerosis’) AND ‘FUS’ AND (‘mouse’ OR ‘mice’)). ALS, amyotrophic lateral sclerosis; SOD1, superoxide dismutase 1;TDP-43, Transactive response DNA binding protein; FUSF, Used in Sarcoma.

The choice to focus on SOD1 mice was based on the predominant role of this model in animal-based research into ALS (see figure 2).

**Figure 3:**
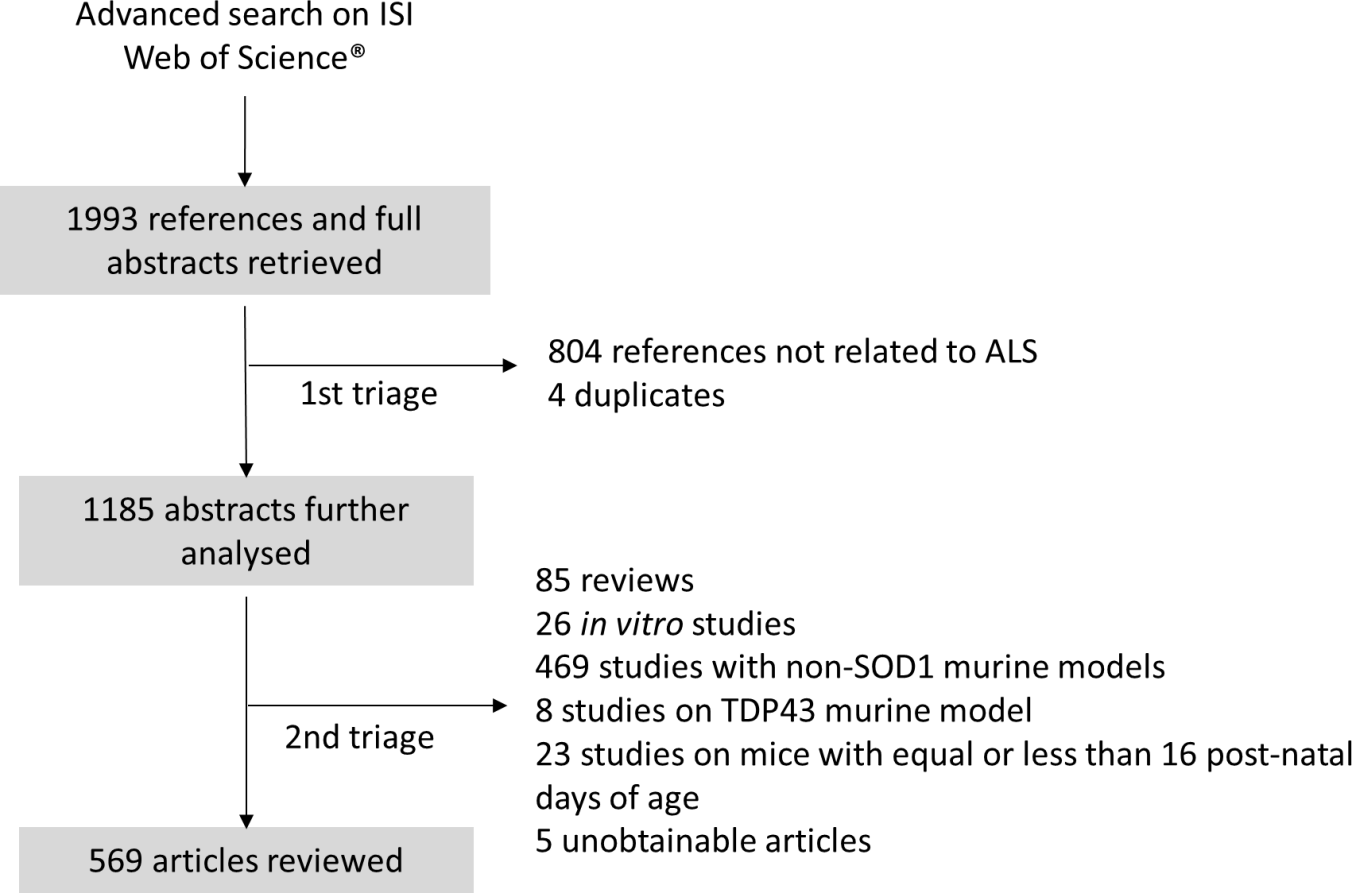
Triage process. The first triage step involved reading each of the 1993 abstracts and excluding all papers that were not related to ALS. The second triage step excluded all papers that did not report original research with SOD1 models of the disease. ALS, amyotrophic lateral sclerosis; SOD1, superoxide dismutase 1; TDP-43, Transactive response DNA binding protein

The search was performed in February 2013 for scientific articles from 2009 and 2011 (after the first and second conferences, respectively), in August 2013 for scientific articles from 2005 (before the two conferences), in September 2014 for scientific articles from 2013, in November 2016 for scientific articles from 2015, and in February 2017 for scientific articles from 2007. After the triage process, illustrated in figure 3, 569 full-text articles remained for analysis: 77 from 2005, 81 from 2007, 84 from 2009, 106 from 2011, 115 from 2013, and 106 from 2015 figure 4.

**Figure 4:**
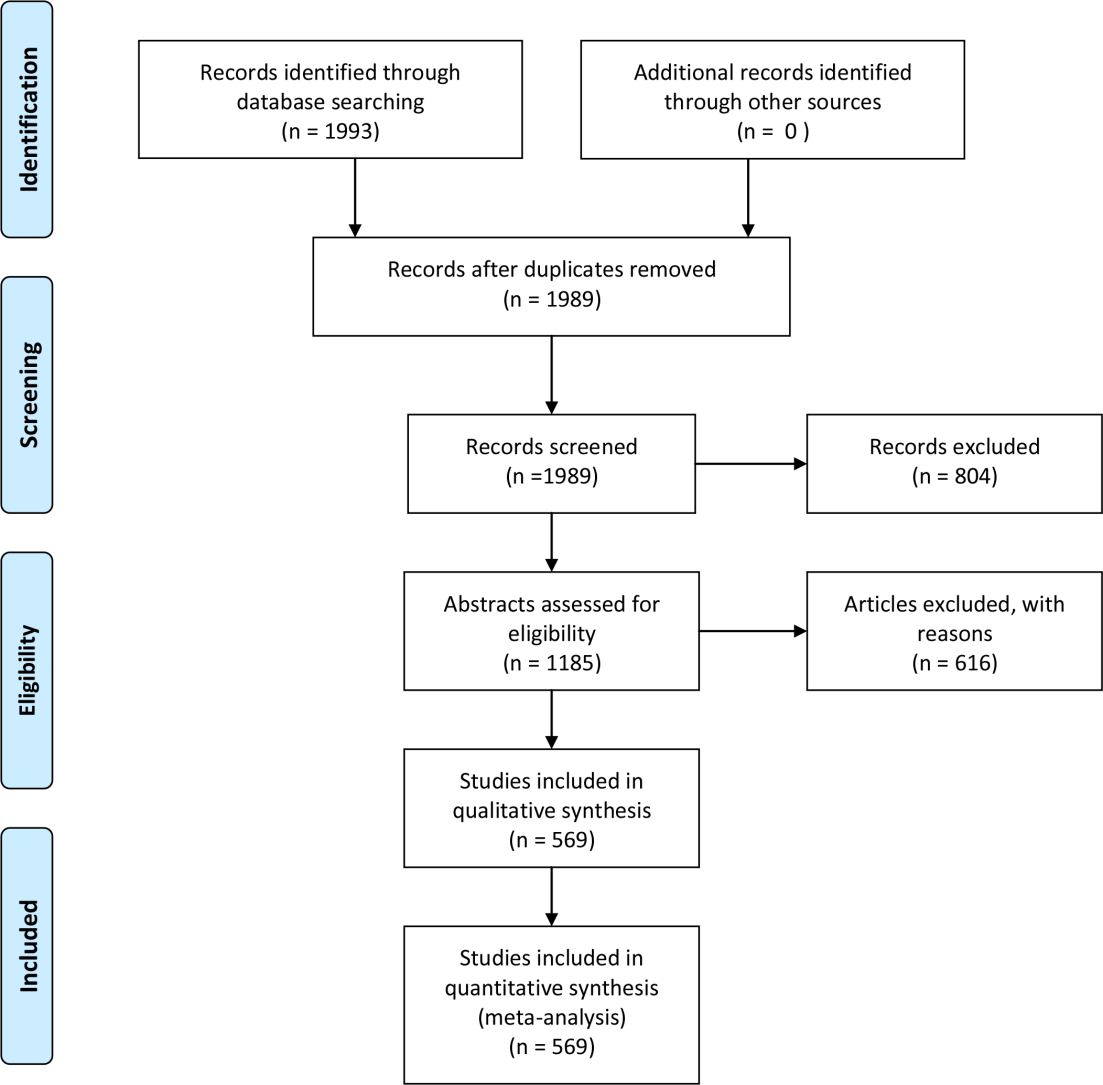
Flow diagram. From Moher D, Liberati A, Tetzlaff J, Altman DG, The PRISMA Group (2009). Preferred Reporting Items for Systematic Reviews and Meta-Analyses: The PRISMA Statement. *PLoS Med* 6(6): e1000097. doi:10.1371/journal.pmed1000097.

### Data collection

Each published study was categorised as either a ‘preclinical’ (ie, carried out ‘to evaluate a drug for use in humans’) or ‘proof-of-concept’ (ie, aiming ‘to elucidate the mechanism of the disease’), according to the suggested classification for animal studies on ALS.^2 38^ Thus, papers reporting outcomes of drug tests in animal models to inform of their therapeutic value for humans were classified as ‘preclinical’, whereas those reporting studies with the primary goal of deciphering a mechanism of the disease without an immediate application to therapeutic approaches in humans—regardless of using a drug as an investigational tool—were classified as ‘proof-of-concept’. Table 1 describes the information retrieved regarding regulatory compliance, animal models, experimental design and animal welfare. This information was retrieved through careful reading of the full papers and logged into a spreadsheet.

**Table 1:** Data retrieved

Category	Items	Description/Observations
Regulatory compliance	Ethical approval	Studies explicitly reported to be approved by a committee/authority.
	Guideline compliance	Articles that did not report having experimental protocols ethically approved by an institution or national entity, but reported that some kind of guidelines for use and care of laboratory animals was followed.
Animal models	Genetic background	When available.
	Sex	Four options: male, female, both or not reported. For *both*, information on whether studies were balanced for gender was retrieved.
	Number of transgene copies	When available.
Experimental design	Group size	Mean group size, based on the available information.
	Randomisation	Studies explicitly reporting assigning animals to groups randomly.
	Blinding	Studies explicitly reporting blinding of observers to experimental groups.
	Non-transgenic littermate control	Studies explicitly reporting the use of non-transgenic littermates as control.
	Splitting littermates into groups	Studies explicitly reporting that littermates were split into groups.
	Housing and husbandry conditions	Reporting information regarding temperature, humidity, light of the room where animals were kept, and cage size and number of animals per cage.
Animal welfare/procedures	Severity	Described in table 2.
	Refinement	Relevant refinements to minimise suffering and distress, such as housing adaptations.
	Euthanasia method	Euthanasia methods were divided into the following categories: ‘Under anaesthesia’ (including anaesthetic overdose), ‘CO_2_ asphyxiation’, ‘Other’, ‘Not reported’ and ‘Not performed’.

A description of the information collected from revised papers is presented for each item

CO2, carbon dioxide.

**Table 2:** Severity scale for ALS studies on transgenic mice with a mutant SOD1 gene

Severity	Description	Welfare issues during this stage
Level 1	Animals euthanised prior to disease onset, which is characterised by progressive weight loss or hind limb tremors.	No overt motor dysfunction. Phenotype is subclinical. Loss of motor function can be detected using rotarod or running wheels, but does not interfere with normal behaviour.
Level 2	Studies terminated at an early stage of disease: animals present trembling and weakness in hind limbs (by approximately 75 days) and mild body weight loss.	Minor. Loss of motor function can be detected using rotarod or running wheels, but has little interference with normal behaviour.
Level 3	Experiments terminated when animals are no longer able to reach food hopper or bottle spout. This occurs when animals reach a moderate (gait abnormalities and weakness) to severe (hind limb paralysis) stage of motor impairment (usually at 120–125 days).	Medium. Loss of motor function and body weight can be detected by monitoring (eg, by a clinical score sheet) and by checking self-righting ability. Refinement measures to address these welfare issues include provision of softer bedding material (eg, sawdust), elongated bottle spouts and mashed food on the cage floor.
Level 4	Animals euthanised after losing the ability to right themselves within 10–30 s after being laid on either side (one or both) or when percentage of weight loss reaches 15%–20% of peak body weight (usually at 130–140 days).	Major. Animals show severe locomotor impairment. Refinement as described for level 3.
Level 5	Animals are euthanised when reaching a moribund stage (complete paralysis) or allowed to die spontaneously.	Severe. At this stage, animals are unable to move, eat or drink. Animals which are not euthanised will die as a result of respiratory failure.

Each severity level exemplified from the most commonly used B6.Cg-TgN-(SOD1G93A) G1H mouse. Classification was based on the most severe endpoint used in each publication.

ALS, amyotrophic lateral sclerosis; SOD1, superoxide dismutase 1.

The review protocol was defined prior to data collection. No modifications to data collection methods were made during the research, but the period to be covered was extended to include publication year 2015. Data extraction was carried out by JGF, with support from NHF, AJG and IASO for disambiguation. Blinding was not possible as access to the full paper was required in order to retrieve information.

For severity assessment, a scale was devised based on the specific characteristics of the ALS models and their progressive disease phenotype (table 2). The ALS models used in the reviewed studies express diverse mutant forms of the *SOD1* gene. The onset of disease for these models is generally characterised by weakness and tremors of the hind limbs, together with a mild loss of body weight. Disease progression leads to paralysis of the hind limbs, followed by complete paralysis (eg, figure 3 in ref 39), accompanied by increased difficulty in eating, drinking and swallowing.^40 41^ Mice die of respiratory failure due to paralysis of the diaphragm.^8^ Age of onset and death, as well as the interval between them, vary depending on the mutation of the amino acid and codon, for example in ref 42, number of copies of transgene, for example in ref 43, and genetic background.^4^ For instance, the overexpressing SOD1G93A Line Gur 1H (B6SJL hybrid) presents with an early onset of overt motor symptoms (3–4 months) and moderate rate of progression (3 weeks from onset to death),^44^ whereas the highly expressing SOD1G85R Line 148 presents with later onset (7.5 months) and faster disease progression (2 weeks from onset to death).^45^ Also, factors such as the animal supplier (eg, refs 46 47), inhouse breeding^48^ and crosses with other non-SOD1 models (eg, SOD1 mice crossed with gene-specific knockout mice^49^) are further sources of variability.

Maximum estimated severity was classified according to a five-level scale (table 2). Scoring was based on the estimated clinical state of animals at the most advanced stage of disease progression they were allowed to reach. Studies in which information was insufficient to draw conclusions about the level of severity were classified as ‘undetermined’. This severity scale was developed building on previous work from members of this team (NHF, IASO), developed for classifying studies on mouse models of Huntington’s disease (table 2 in ref 5), together with our own (AJG) experience with mutant SOD1 mouse models and literature. For purposes of statistical analysis, the severity scale was reduced to a binary scale (‘low’=severity up to level 4; ‘high’=level 5 severity). The choice for above level 4 severity as a cut-off point was based on its status as a ‘standard endpoint’ in published ALS guidelines,^2 38^ whereas full paralysis or spontaneous death exceeds this standard endpoint, as well as the legally recommended endpoints in many countries, including the European Union Member States.

### Methodological Standards Reporting and Regulatory Compliance Reporting scores

For each reviewed publication, data were collected on a number of items which all contributed with information about the reporting quality of the paper. For the analysis, we brought these items together into two scores, hence generating for each paper two comprehensive measures for reporting quality, one on methodological standards and one on regulatory compliance. We then used regression analysis to investigate how the two scores (dependent variables) were related to publication year, type of study, country of origin and journal (explanatory or predictor variables), as outlined in detail in the following. Based on the regression models, it is possible to predict how the dependent variables would have changed with changes in the explanatory variables. In contrast to, for example, correlation, the regression analysis takes into account all the explanatory variables that were included in the models, that is, the estimated association between a score and one of the explanatory variables is independent of the values of the other explanatory variables considered. In that way, spurious associations caused by the relationships between the explanatory variables in the data can be avoided.

The two scores were formed as weighted sums of separate sets of items. The Methodological Standards Reporting (MSR) score was constructed as the weighted sum of the items *sampsize*, *climate*, *cagesize*, *nmice*, *sex*, *copies* and *genetic* (which refer to important research parameters in animal experimentation and in ALS research in particular) and the items *random*, *blinded*, *control*, *sibsplit* and *exclus* (associated with general good practices in the design of animal experiments and published recommendations for ALS studies). Greater weight (1.5 vs 1) was attributed to items which are also part of the ALS guidelines. Table 3 describes these items, their attributed weight in the MSR score and the absolute number and percentage of papers reporting this information, divided by the type of study.

**Table 3:** List of items integrated in the MSR and RCR scores for preclinical (n=108) and proof-of-concept (n=461) animal studies on ALS reporting this information

Reported information	MSR score		‘Proof-of-Concept’ (n=461)		‘Preclinical’ (n=108)	
	Score item	Score weight	Absolute number	%	Absolute number	%
Relevant animal research variables						
Group size	*sampsize*	1.5	368	79.8	106	98.1
Environment: light, temperature, humidity (fully or partially reported)	*climate*	1	123	26.7	42	38.9
Cage size	*cagesize*	1	1	0.2	2	1.9
Mice per cage	*nmice*	1	26	5.6	15	13.9
Sex of the animals	*sex*	1.5	223	48.4	71	65.7
Number of transgene copies	*copies*	1.5	286	62.0	80	74.1
Genetic background	*genetic*	1.5	349	75.7	92	85.2
Measures to reduce ‘noise’ and bias in experiments						
Animals randomised to treatment groups	*random*	1	28	6.1	47	43.5
Observers blinded to treatment	*blinded*	1.5	94	20.4	52	48.1
Non-transgenic littermate controls used	*control*	1	150	32.5	39	36.1
Splitting littermates into groups	*sibsplit*	1	28	6.1	31	28.7
Reason for exclusion of animals is reported	*exclus*	1	2	0.4	6	5.6

	RCR score		‘Proof-of-Concept’ (n=461)		‘Preclinical’ (n=108)	
Self-reported compliance with laws and regulations	*comply*	1	98	21.3	28	25.9
Project approval reported	*protocol*	1	315	68.3	66	61.1
Refinement measures (eg, to aid, feed and hydrate)	*refine*	1	29	6.3	14	13

The score for each variable is provided (MSR score ranging from 0 to 12.5, and RCR score ranging from 0 to 3). Greater weight (1.5 vs 1) was attributed to items which are also part of the ALS guidelines. For purposes of statistical modelling, RCR (only including items *comply*, *protocol* and *refine*) was later simplified to a binary variable RCRb, coded as 1 for RCR values 2–3 and as 0 for RCR values 0–1.

ALS, amyotrophic lateral sclerosis; MSR, Methodological Standards Reporting; RCR, Regulatory Compliance Reporting.

The Regulatory Compliance Reporting (RCR) score was originally constructed from the items *comply*, *protocol*, *severity* (turned into a binary classification) and *refine*. For purposes of statistical modelling, the final version of this score (RCRb) included *comply*, *protocol* and *refine* and was coded as 1 when the sum of these was 2–3, and as 0 when the sum was 0–1.

MSR and RCRb were modelled statistically to estimate the effects of publication year (2005, 2007, 2009, 2011, 2013 or 2015), study type (preclinical or proof-of-concept), country of origin (15 categories), journal (17 categories) and severity (low or high), simultaneously accounting for all the explanatory variables in the models. Countries contributing with less than 12 papers and journals contributing with less than 6 papers were combined into separate categories, denoted ‘Other’. MSR was modelled using linear regression and RCRb by logistic regression. Logistic regression is appropriate for binary dependent variables (assuming a linear relationship of the log-odds of the dependent variable with the explanatory variables). The results of a logistic regression can be expressed as the odds of a positive value of the dependent variable at one level of a categorical explanatory variable relative to the odds at another level (the ORs), or the probability of a positive dependent variable at any given level of the explanatory variables. All first-order interaction effects (combined effects of two explanatory variables at a time) were tested and included if significant.

Predictive marginal means were calculated, showing predicted values of MSR and probabilities of RCR being above 1 for different publication years, study types and countries of origin. In each case, the marginal means assumed remaining variables in the models to have their observed values. Both models were checked using the Pregibon link test^50^ and by examining standardised residuals, looking for model misspecification and extreme values. The MSR model was also checked with the Breusch-Pagan/Cook-Weisberg test for heteroscedasticity^51^ (variability differing between parts of the data), the Ramsey regression specification error test for omitted variables,^52^ and the RCRb model by examining delta-betas to identify particularly influential observations. The proportion of the total variation in MSR and RCRb that could be explained by differences between countries or journals was determined by running empty mixed models with country and journal, respectively, as a random effect, and calculating the intraclass correlation coefficients. The justification for weighting the items composing MSR was checked by modelling an alternative score formed without weighting. The differences between years and countries remained virtually unchanged, although the unweighted score values were generally lower.

The association between MSR and RCR scores was estimated using Spearman rank correlation, which is suitable for non-normally distributed data. A total of 490 observations could be used. Overall MSR mean±SD was 5.69±2.39. RCR assumed the values of 0 (n=48), 1 (n=103), 2 (n=309) or 3 (n=30), resulting in 69% of the observations having values above 1. The number of observations per level of year, study type, country, journal and severity is shown in table 4.

**Table 4:** Distribution of observations across levels of independent variables included in models of Methodological Standards Reporting and Regulatory Compliance Reporting indices in 490 amyotrophic lateral sclerosis studies

Variable	Level	n	Percentage
Year	2005	77	13.58
	2007	81	14.29
	2009	83	14.64
	2011	106	18.69
	2013	115	20.28
	2015	105	18.52
Study type	Concept	460	81.13
	Preclinical	107	18.87
Country	Australia	14	2.47
	Belgium	14	2.47
	Canada	29	5.11
	China	28	4.94
	France	18	3.17
	Germany	25	4.41
	Israel	18	3.17
	Italy	50	8.82
	Japan	65	11.46
	Other	43	7.58
	South Korea	25	4.41
	Spain	15	2.65
	Sweden	12	2.12
	UK	23	4.06
	USA	188	33.16
Journal	*Brain*	9	1.59
	*Brain Res*	20	3.53
	*Eur J Neurosci*	8	1.41
	*Exp Neurol*	31	5.47
	*Front Cell Neurosci*	12	2.12
	*Hum Mol Gen*	24	4.23
	*J Biol Chem*	19	3.35
	*J Neurochem*	15	2.65
	*J Neuroinflamm*	9	1.59
	*J Neurosci*	20	3.53
	*J Neurosci Res*	9	1.59
	*Mol Neurodegener*	8	1.41
	*Neurobiol Dis*	34	6.00
	*Neurosci*	15	2.65
	Other	271	47.80
	*PLOS One*	45	7.94
	*Proc Natl Acad Sci USA*	18	3.17
Severity	1	14	2.5
	2	20	3.5
	3	46	8.1
	4	346	60.8
	5	64	11.2
	Inconclusive	79	13.9

The data were analysed in Stata/IC V.13.1 and IBM SPSS V.23.0. Each article was regarded as the experimental unit and the level of significance for all tests was 0.05.

## Results

### Quality of research and reporting

The quality of methodological standards and of reporting is crucial to avoid bias and achieve reliable, repeatable and translatable research results. We measured this through the MSR score and also looked at specific research parameters individually.

#### MSR score

The 12 items that comprise the MSR score represent 7 relevant experimental variables and 5 measures for reducing bias in animal experiments. Higher scores mean better reporting and implementation of good practices in the design of ALS animal studies.

**Figure 5:**
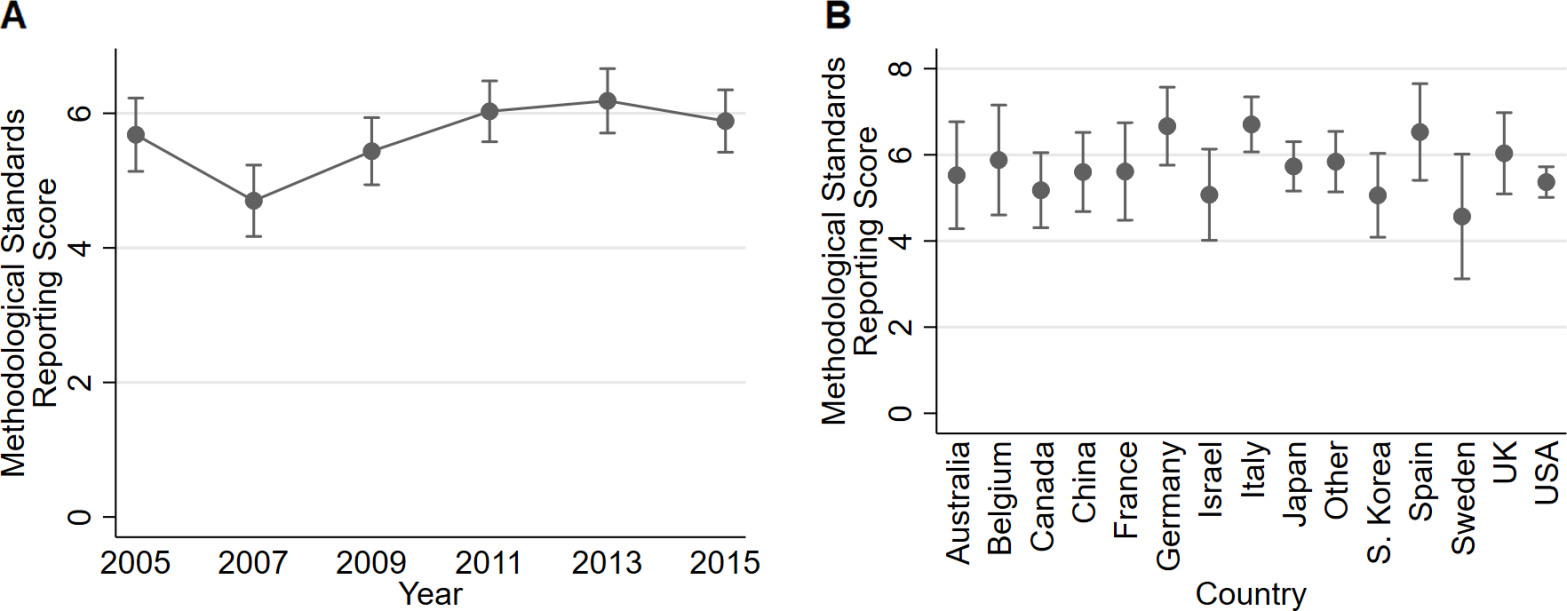
Predictive marginal means (predicted score values) ±95% CI of publication year (A) and country (B) based on a model of an MSR score in 487 ALS studies. According to the linear regression model, MSR could be expected to be lower in 2007 than in 2005, but higher in 2009, 2011, 2013 and 2015 than in 2007. No significant interactions were found (eg, between country and year). According to the R-square statistics, the model explained 25% of the total variation in MSR. ALS, amyotrophic lateral sclerosis; MSR, Methodological Standards Reporting.

MSR was significantly affected by year and study type (joint *F*-test p=0.0015 and p<0.0001, respectively). Compared with 2005, the logistic regression model predicted a lower MSR for 2007. However, the subsequent years (2009, 2011, 2013 and 2015) were all predicted to be higher than 2007, with a consistent and unbroken increasing trend until 2013 (figure 5). In 2013, MSR was predicted to be 1.5 units higher than in 2007 (p<0.0001). The model also predicted a higher MSR for preclinical studies than for proof-of-concept studies (marginal mean of 7.28 and 5.26, respectively). Model diagnostics showed that linear regression was justified and the model fit was excellent. Table 5 shows the complete MSR model results.

**Table 5:** Model estimates of an MSR index, from the 487 ALS studies that could be used

Variable	Level	Coefficient	SE	P value	95% CI
Intercept	–	6.96	0.572	<0.0001	5.83 to 8.08
Year	2005	0.981	0.387	0.011	0.222 to 1.74
	2007	0	–	0.0015	–
	2009	0.736	0.369	0.047	0.0110 to 1.46
	2011	1.33	0.355	<0.0001	0.631 to 2.03
	2013	1.49	0.375	<0.0001	0.748 to 2.22
	2015	1.18	0.370	0.001	0.458 to 1.91
Study type	Preclinical	0	–	<0.0001	–
	Proof-of-concept	−2.02	0.249	<0.0001	−2.51 to −1.53
Country	Australia	0.159	0.661	0.81	−1.14 to 1.46
	Belgium	0.512	0.676	0.45	−0.817 to 1.84
	Canada	−0.188	0.479	0.70	−1.13 to 0.754
	China	0.235	0.508	0.64	−0.764 to 1.23
	France	0.245	0.607	0.69	−0.948 to 1.44
	Germany	1.30	0.493	0.009	0.330 to 2.27
	Israel	−0.294	0.570	0.61	−1.41 to 0.827
	Italy	1.34	0.377	<0.0001	0.598 to 2.08
	Japan	0.365	0.343	0.29	−0.308 to 1.04
	Other	0.474	0.402	0.24	−0.315 to 1.26
	South Korea	−0.307	0.529	0.56	−1.35 to 0.733
	Spain	1.16	0.604	0.055	−0.0249 to 2.35
	Sweden	−0.798	0.756	0.29	−2.28 to 0.688
	UK	0.668	0.514	0.19	−0.342 to 1.68
	USA	0	–	0.025	–
Journal	*Brain*	−1.59	0.859	0.065	−3.27 to 0.100
	*Brain Res*	−1.45	0.641	0.024	−2.71 to −0.187
	*Eur J Neurosci*	−1.12	0.868	0.20	−2.83 to 0.581
	*Exp Neurol*	−0.993	0.573	0.084	−2.12 to 0.133
	*Front Cell Neurosci*	−0.251	0.904	0.78	−2.03 to 1.52
	*Hum Mol Gen*	−1.50	0.589	0.011	−2.66 to −0.343
	*J Biol Chem*	−1.88	0.662	−0.005	−3.19 to −0.583
	*J Neurochem*	−0.213	0.714	0.77	−1.62 to 1.19
	*J Neuroinflamm*	−1.37	0.886	0.12	−3.11 to 0.369
	*J Neurosci*	−1.05	0.617	0.090	−2.26 to 0.163
	*J Neurosci Res*	−1.14	0.897	0.20	−2.90 to 0.619
	*Mol Neurodegener*	0.0040	0.909	0.99	−1.78 to 1.79
	*Neurobiol Dis*	−1.53	0.533	0.004	−2.58 to −0.481
	*Neurosci*	−0.479	0.668	0.47	−1.79 to 0.834
	Other	−1.11	0.385	0.004	−1.86 to −0.348
	*PLOS One*	0	–	0.14	–
	*Proc Natl Acad Sci USA*	−1.88	0.672	0.005	−3.20 to −0.560
Severity	Low	0	–	0.80	–
	High	−0.0767	0.306	0.80	−0.679 to 0.525

ALS, amyotrophic lateral sclerosis; MSR, Methodological Standards Reporting.

#### Reporting of relevant research parameters

Some research parameters were very seldom reported, for example, numbers of animals per cage (7.2%, 41/569), cage size (0.5%, 3/569) and exclusion of animals (1.4%, 8/569). Measures in guideline recommendations to reduce bias in ALS research were mostly not reported, including splitting littermates to treatment groups (10.4%, 59/569), use of non-transgenic littermates as controls (33.2%, 189/569), as well as measures of broader application, such as random assignment of animals to treatments (13.2%, 75/569) or blinding of observers (25.7%, 146/569). By contrast, numbers of transgene copies and genetic backgrounds of animals were reported in the majority of papers.

Of papers reporting sex (n=297), 54.2% (161/297) described studies using mice of both sexes, while 29.0% (86/297) used only males and 16.8% (50/297) used only females. Reporting of sex rose steadily from 2005 (39.0%, 30/77) to 2015 (69.8%, 74/106).

Regarding the chosen genetic background of animals used for preclinical studies (n=108), 76% (70/92) of those reporting this parameter generated experimental animals using a cross between mice hemizygous for the SOD1 mutant gene and C57/SJL outbred strains.

**Figure 6:**
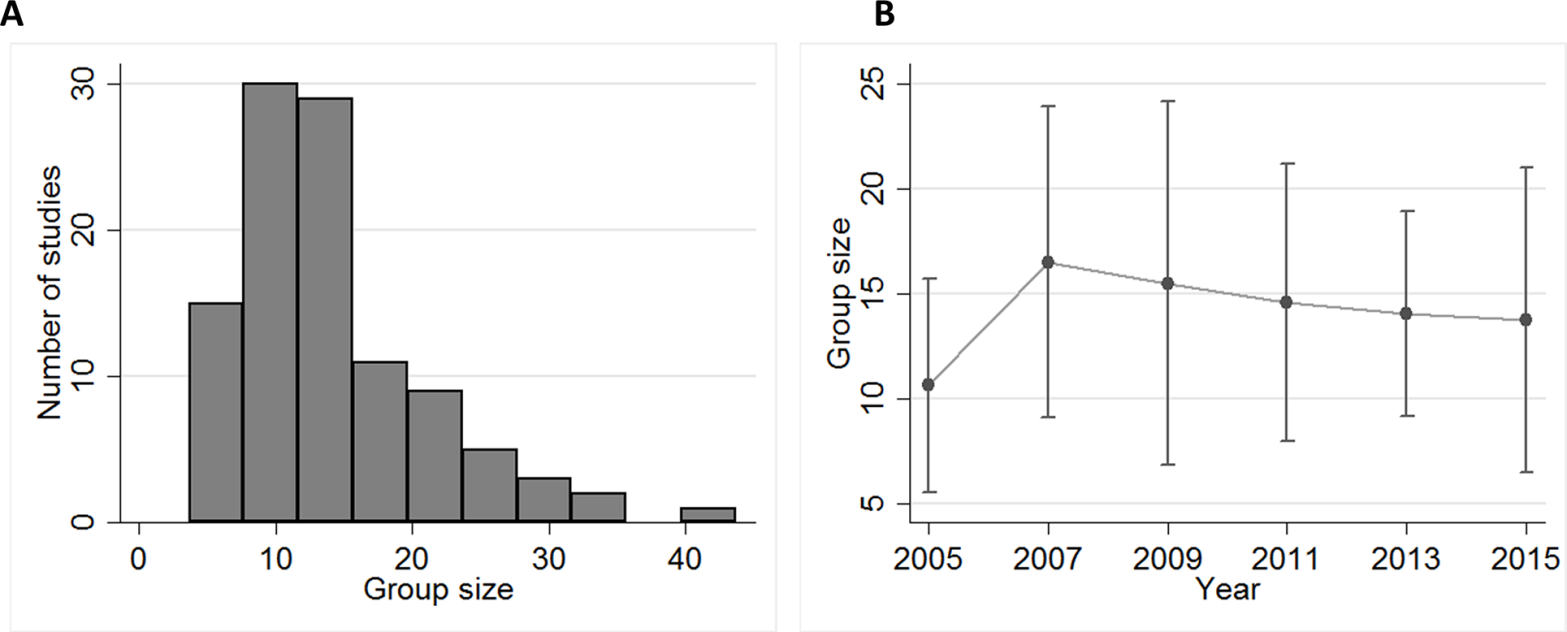
Group size. Histogram of mean group size in 105 preclinical studies reporting this parameter (A) and for each of the years analysed (yearly mean±1 SD) (B).

Only 10 studies (6 proof-of-concept studies and 4 preclinical studies) from 2007, 2009, 2011, 2013 and 2015 justified the number of animals used per group. However, of these, only six gave clear justifications (five justified the group size by a power analysis and the other by the size of groups proposed in ALS guidelines).^2 38^ On the other hand, group size was reported in 83.3% (474/569) of ALS papers, and more so in the preclinical studies subsample (figure 6).

Of the 569 papers reviewed, 38% (214/569) did not report the method for killing animals despite the fact that in 91% (195/214) of these terminal procedures requiring anaesthesia for ethical and practical reasons were identified (eg, transcardial perfusion fixation). The most commonly used euthanasia method—of the papers reporting this information—was anaesthetic overdose or the use of another method under anaesthesia (86%, 317/367), while other methods such as carbon dioxide asphyxiation (7%, 26/367) or others such as decapitation or cervical dislocation (7%, 24/367) were seldom used. Very few studies (15/569) were identified as not performing euthanasia of any kind. The remaining 21 articles were deemed ‘inconclusive’, for neither reporting euthanising animals at any point nor reporting deaths.

### Regulatory compliance and estimated severity

For public confidence in research, it is important that research with animals is carried out according to standards set by legislation and in line with the principles of the 3Rs. We measured such compliance through the RCR score and also looked at specific research parameters individually.

#### RCR score

**Figure 7:**
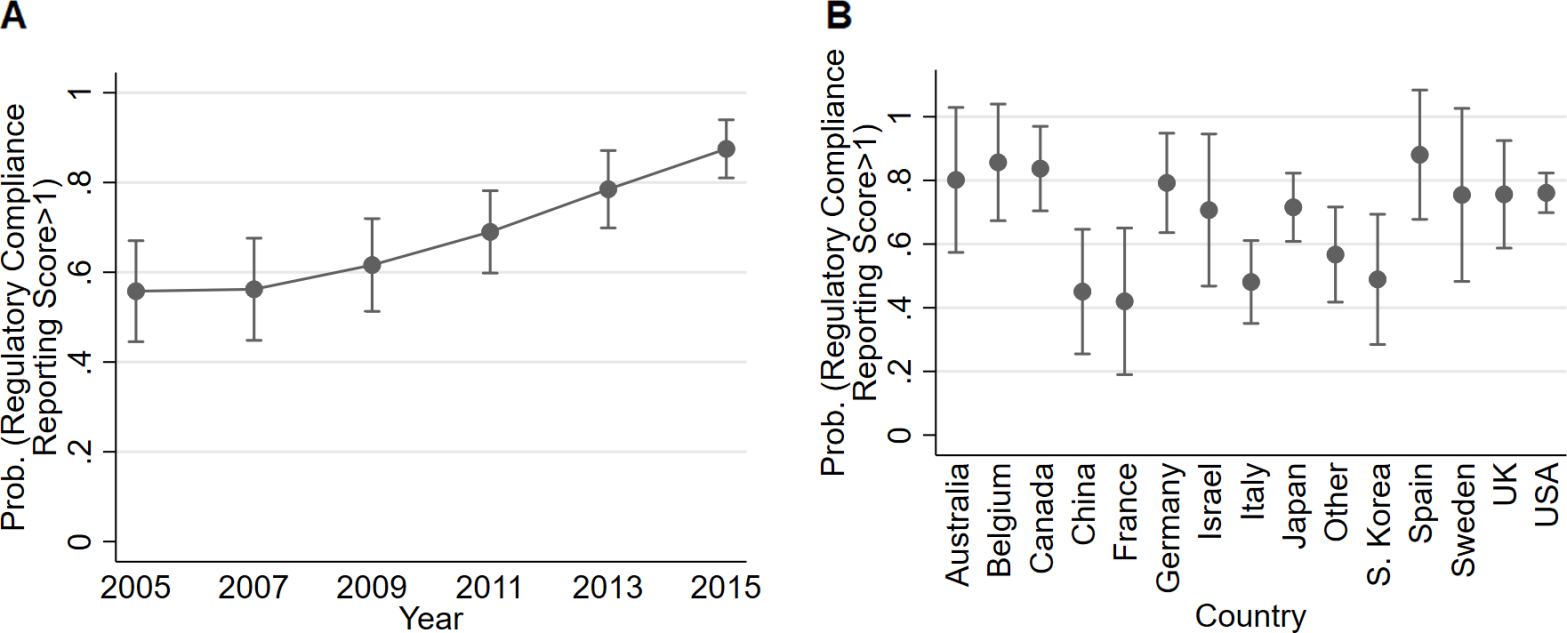
Predictive marginal means (predicted probabilities of values >1) ±95% CI of publication year (A) and country (B) based on a model of an RCR score in 490 ALS studies. The probability of an RCR score above 1 was higher in 2013 and 2015 than in 2005. China, France, Italy and South Korea appeared to have comparatively low probabilities, while for example Spain, Belgium and Canada had somewhat high probabilities. No significant interactions were found. The pseudo R-square statistics indicated that the model explained 16% of the total variation in the data. ALS, amyotrophic lateral sclerosis; RCR, Regulatory Compliance Reporting.

The RCR score, which measures to what extent compliance with legislation and approval of animal experiments are reported in published papers, shows an overall improvement in the reporting over the time period under study (joint χ^2^ p<0.001; figure 7). The estimated odds of RCR >1 was 7.1 times higher in 2015 than in 2005 (p<0.0001). RCR did not differ between journals or between proof-of-concept and preclinical studies, but was affected by country (figure 7). Studies with high severity seemed to have higher odds of high RCR values (p=0.027). Model diagnostics showed that logistic regression was justified. Table 6 shows the RCR model results.

**Table 6:** Model estimates of an RCR index in 490 ALS studies

Variable	Level	OR	SE	P value	95% CI
Intercept	–	3.62	2.34	0.047	1.02 to 12.9
Year	2005	1	–	0.0001	–
	2007	1.02	0.397	0.96	0.476 to 2.19
	2009	1.33	0.502	0.46	0.631 to 2.79
	2011	1.94	0.728	0.077	0.931 to 4.05
	2013	3.43	1.41	0.003	1.53 to 7.67
	2015	7.07	3.16	<0.0001	2.95 to 17.0
Study type	Concept	1.37	0.369	0.24	0.812 to 2.32
Country	Australia	1.31	1.11	0.75	0.250 to 6.85
	Belgium	2.02	1.73	0.41	0.375 to 10.9
	Canada	1.71	1.00	0.36	0.545 to 5.38
	China	0.199	0.111	0.004	0.0671 to 0.592
	France	0.171	0.109	0.006	0.0489 to 0.599
	Germany	1.23	0.710	0.72	0.395 to 3.81
	Israel	0.726	0.521	0.66	0.178 to 2.96
	Italy	0.231	0.0916	0.000	0.106 to 0.503
	Japan	0.763	0.282	0.47	0.369 to 1.58
	Other	0.352	0.152	0.016	0.151 to 0.821
	South Korea	0.241	0.134	0.010	0.0810 to 0.716
	Spain	2.54	2.76	0.39	0.303 to 21.3
	Sweden	0.960	0.843	0.96	0.172 to 5.36
	UK	0.971	0.554	0.96	0.318 to 2.97
	USA	1	–	0.010	–
Journal	*Brain*	0.266	0.256	0.17	0.0406 to 1.75
	*Brain Res*	0.206	0.153	0.033	0.0482 to 0.880
	*Eur J Neurosci*	0.772	0.813	0.81	0.0979 to 6.09
	*Exp Neurol*	0.316	0.226	0.11	0.0782 to 1.28
	*Front Cell Neurosci*	0.134	0.150	0.072	0.0150 to 1.20
	*Hum Mol Gen*	0.427	0.318	0.25	0.0992 to 1.84
	*J Biol Chem*	0.257	0.199	0.079	0.0566 to 1.17
	*J Neurochem*	0.196	0.158	0.044	0.0404 to 0.954
	*J Neuroinflamm*	0.780	1.00	0.85	0.0632 to 9.63
	*J Neurosci*	0.119	0.0879	0.004	0.0279 to 0.506
	*J Neurosci Res*	0.302	0.314	0.25	0.0394 to 2.32
	*Mol Neurodegener*	0.131	0.129	0.039	0.0189 to 0.903
	*Neurobiol Dis*	0.250	0.170	0.041	0.0661 to 0.947
	*Neurosci*	1.66	1.97	0.67	0.161 to 17.1
	Other	0.368	0.204	0.071	0.125 to 1.09
	*PLOS One*	1	–	0.41	–
	*Proc Natl Acad Sci USA*	0.382	0.323	0.26	0.0730 to 2.00
Severity	Low	0	–	0.027	–
	High	2.21	0.790	0.027	1.10 to 4.45

ALS, amyotrophic lateral sclerosis; RCR, Regulatory Compliance Reporting.

Over the entire period, most papers (67.0%, 381/569) reported that studies had been appraised and approved by a third party (eg, ethics committee, competent authority), with only 10.9% (62/569) not reporting any kind of regulatory compliance. By 2015, all papers were found to have some type of statement on regulatory compliance, most of which (83%) referring to prior ethical approval of research protocols.

The correlation between MSR and RCR was weak but highly significant (Spearman r=0.21, p<0.0001), indicating that papers with high scores for methodological standards were somewhat more likely to also score highly for regulatory standards.

#### Severity and refinement measures

**Figure 8:**
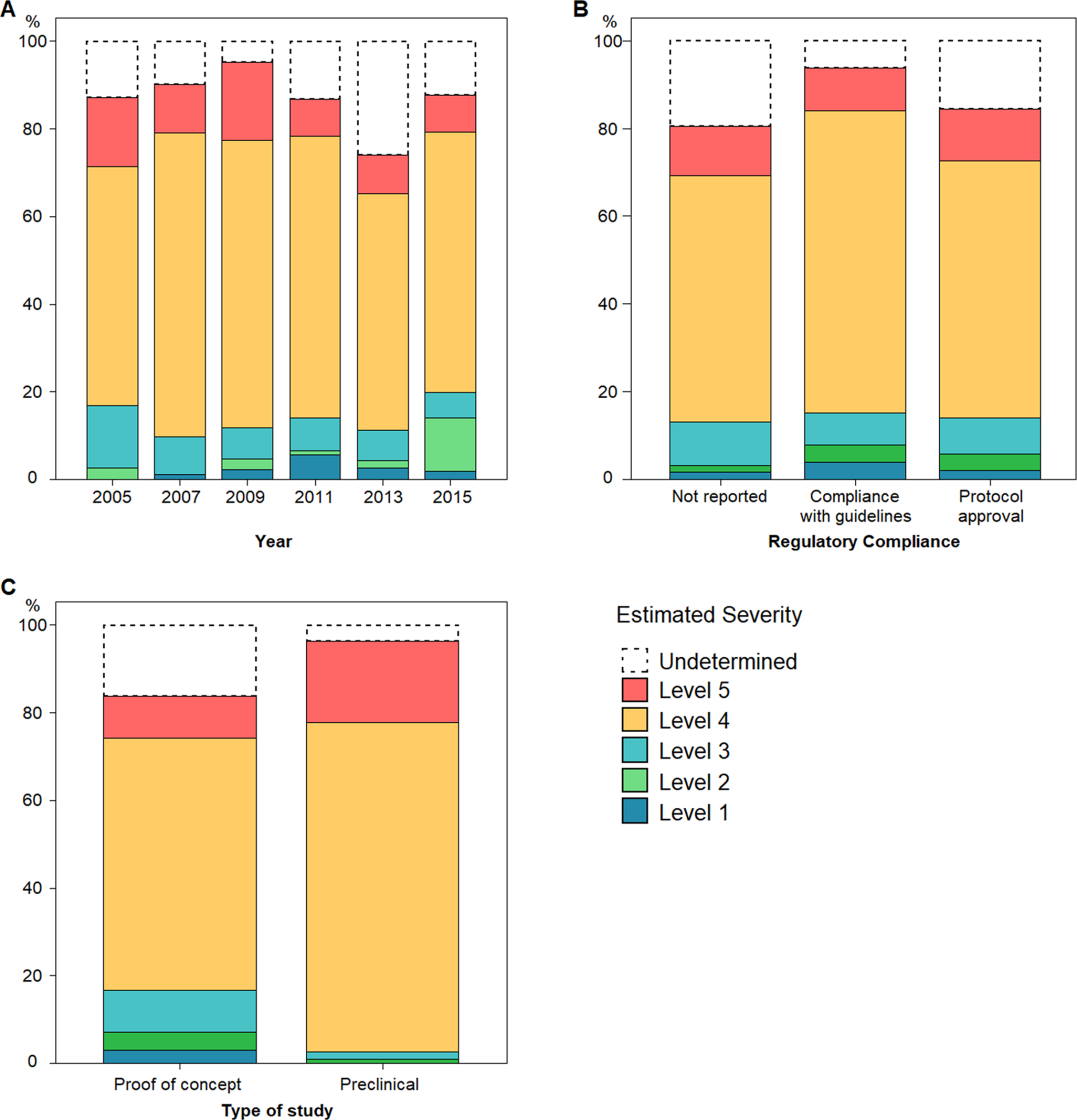
Severity classification of studies (n=569). (A) Percentage of studies, by year, classified into each of the five levels of our severity scale, as well as those of ‘undetermined’ severity due to insufficient information (n=77 in 2005; n=81 in 2007; n=84 in 2009; n=106 in 2011; n=115 in 2013; n=106 in 2015). (B and C) Percentage of studies classified into each of the five levels, according to, respectively, reported regulatory compliance status (n=62, not reported; n=126, guidelines followed; n=381, protocol approval) and type of study (n=461, proof-of-concept studies; n=108, preclinical studies).

We have found in previous systematic reviews^5 6 53^ that self-reported compliance with regulations may not necessarily affect the severity of the experiments being conducted. To test whether actual experimental practice has changed over the study period, we classified the severity of each study according to the criteria in table 2. The majority of publications (60.7%, 346/569) included experiments at level 4 severity (figure 8A). Of the 64 studies classified as level 5 (allowing animals to die of disease progression or to reach complete paralysis), 89% reported regulatory compliance (70% ethical approval from a national authority or institutional ethics committee and 19% compliance with relevant legislation or animal use guidelines). However, between those studies that reported regulatory compliance and those that did not, there was no difference in the proportion that were level 5 (χ^2^ (5 df)=2.855, p=0.722) (figure 8B).

On the other hand, we did observe a difference between preclinical and proof-of-concept studies: preclinical studies included a higher proportion of studies within the highest severity categories (77.9% (81/104) classified as level 4 and 19.2% (20/104) as level 5) than did proof-of-concept studies (68.7% (265/386) classified as level 4 and 11.4% (44/386) as level 5). Moreover, no preclinical studies were given a level 1 or level 2 severity (χ^2^ (5 df)=19.593, p=0.001) (figure 8C).

Of studies classified between level 3 and level 5 severity (ie, from which it could be ascertained animals presented overt locomotor impairments), only 9.1% (42/456) described any refinement measures to alleviate suffering (eg, provision of mashed food and adaptation of bedding in later stages of disease progression), which occurred almost exclusively (39/42) in level 4 studies.

Differences in the regulatory landscape between countries imply that *how* animals are treated in biomedical research may depend on *where* these experiments are carried out. The proportion of high severity (level 5) studies differed significantly (χ^2^ (13 df)=35 561, p=0.001) between the 14 most represented countries in our sample, ranging from 40% (8/20) and 41% (7/17) in South Korea and Israel, respectively, to 4% in Canada and China, and even none in Belgium (0/14) and the UK (0/23).

## Discussion

Our analysis, the first of its kind to use specially devised scores encompassing both methodological standards and regulatory compliance reporting (MSR and RCR, respectively) over a 10-year period, suggests three main findings: The first is an overall improvement in both regulatory compliance and methodological and reporting quality across the period assessed. Also, and somewhat as expected, studies classified as ‘preclinical’ scored higher for methodological and reporting quality as compared with more ‘proof-of-concept’ studies. The third finding is that these scores varied widely according to the country in which the first author was based, but not according to the journal publishing the paper.

The improved reporting of regulatory compliance, as expressed in the increase in RCR score across time, is an indicator of widespread increase in reported adherence to animal welfare regulatory requirements. However, this was not reflected in any significant change in the proportion of highly severe (level 5 in our classification scheme) studies or the reporting of refinement measures (in studies where animals showed overt clinical signs). This is in agreement with results from previous systematic reviews of animal research on Huntington’s disease (papers published in 1997–2009)^5^ and tuberculosis (1997–2011).^6^ Also, while ‘preclinical’ studies were more likely to be classified in the higher severity categories, there was no relation between the level of severity and whether papers reported approval of protocols or compliance with regulations, the latter also reflecting previous findings.^5 53^

Only 11.2% of ALS studies were classified at the highest severity level (level 5, ie, including experiments with spontaneous death or euthanasia at a near-death stage, ie, complete paralysis), which is much lower than that found in research using mouse models of Huntington’s disease (38%)^5^ and tuberculosis (66%).^6^ Moreover, most endpoints applied in ALS studies adhered to the same basic criterion for euthanising animals, namely the point at which animals are unable to resume their position if laid recumbent within 10–30 s. This is the primary endpoint proposed in existing guidelines for preclinical ALS^2 38^ and the ALS Treatment Development Institute’s recommendations^28^ (level 4 severity on our scale), suggesting researchers to a great extent act in accordance with published guidance in this respect. However, this endpoint was already broadly used before the publication of the guidelines, suggesting that these reflect common practice at the time of publication.

Applying predefined endpoints is important to prevent the loss of biological samples from animals found dead and for which time of death therefore cannot be defined,^5^ hence maintaining numbers of animals and avoiding loss of statistical power and subsequent inconclusive results. However, from an animal welfare perspective, the current standard endpoint for ALS studies corresponds to an end stage where euthanasia may prevent deaths from respiratory failure, but since they seldom anticipate death by more than a day, or even just a few hours, late-stage endpoints only curtail a small part of animal suffering.^7^ Very late endpoints increase the likelihood that at least some animals will die unsupervised (eg, overnight), while the confounding effect of starvation and dehydration in survival data increases as animals become progressively less able to reach the bottle spout or the food hopper.^54^ At advanced clinical stages, refinements such as providing mashed food on the cage floor, long-spouted water bottles or fluid administration are therefore crucial to avoid unnecessary animal suffering and to improve validity by bringing the model closer to the clinical setting, where late-stage human patients are provided palliative care.^55^ Defining endpoints also needs to take the research purpose into account. In ALS, the mechanisms operating at different stages of the disease are known to be different, principally affecting distal axons at the onset of symptoms, but developing an immune/inflammatory phenotype during the end stages.^56^ Therefore, endpoints relevant to the treatment strategies must be used, particularly when targeting neuroinflammation.

MSR improved over the time period under study. Studies classified as ‘preclinical’ reported methodology in more detail than those deemed ‘proof-of-concept’, consistent with the view that a more rigorous design and execution should be demanded for preclinical studies.^57^ Nevertheless, the checklist provided in the 2010 edition of the guidelines for ALS research sets high methodological standards for both types of studies.^2^ Throughout the period under study, the MSR scores remain below 50% of the maximum score, showing that the overall level of reporting of methodological detail remains substantially below the recommendations in the guidelines.

Only three parameters (genetic background, number of transgene copies and group size) were reported in more than half of the sample, whereas other relevant information, such as housing conditions, randomisation of animals into treatment groups or blinding of researchers, was absent in well over two-thirds of the papers analysed, in line with previous reviews of animal research in the neurosciences.^5 54 58^ Other biological and methodological parameters such as sex (only reported in the majority of papers in the ‘preclinical studies’ subsample) and method of choice for euthanising animals were also largely under-reported. The method used for euthanising animals has both animal welfare implications and scientific relevance, as the method affects biological and histological parameters differently, which can impact the postmortem data collected.^59 60^ The increase in the proportion of articles in our sample reporting sex of the animals is positive, as sex differences^4 61–63^ in the phenotype or response to therapeutic drugs may influence results and be of clinical relevance. However, although ALS guidelines propose the use of both male and female mice, little over half of the studies providing this information reported doing so. Overall, making these and other details on animals and protocol available is central to allowing an adequate interpretation of results and a critical evaluation of their validity, as well as allowing study replication and proper integration of results in systematic reviews and meta-analyses.^31 64^

Sample size was generally well reported, but of those reporting this parameter only a small minority used the 24 per group recommended in the 2010 guidelines.^2^ Furthermore, only three studies clearly justified group size, in agreement with previous reports that this is frequently overlooked, for example refs 31 65. Adequate sample size is paramount to ensure that animals, time and resources are not wasted as a result of underpowering experiments by using too few animals.^66 67^ Noise reduction by genetic standardisation could also help reduce the number of animals needed per study, as the reduced interindividual variability of isogenic strains allows increasing power without requiring more animals^68^ and is indeed mentioned in the 2007 guidelines as a way of reducing variability in drug testing.^38^ Mead and colleagues,^69^ for instance, have shown great consistency of results by using SOD1G93A transgenic mice on an inbred C57BL/6 genetic background, with the added advantage of presenting early indicators of disease progress, allowing for faster and more humane drug screening. Only 11% of the preclinical studies reviewed, however, used a fully inbred background. The use of a single well-characterised model for initial studies can be supported further by independent replication studies in a different disease model.

Most articles did not report random assignment of animals to groups or blinded outcome assessment. This reflects similar data from reviews on the methodological quality of preclinical research on ALS^28 58 70^ and other fields.^31 33 71–73^ This lack of attention to measures to avoid noise and biases in animal experiments is cause for concern, given their role in improving the reliability of results, as well as the translational value of preclinical research.^16 24 33 67 71^ While it cannot be excluded that in some cases blinding and randomisation were applied but not reported, one might expect that researchers carrying out well thought out and planned experiments would state such measures, since this strengthens their results and conclusions. There is ample evidence for many areas^32 33 73–75^ that published studies which do not report measures to minimise bias (ie, blinding, randomisation and allocation concealment) tend to present an exaggerated estimate of the therapeutic effect of experimental drugs. This is particularly relevant in the light of the ongoing discussion of why promising preclinical results of candidate drugs for ALS have not translated into the clinic. Although the disappointing outcomes of clinical trials apparently contradict the promising preclinical results that elicited them, they may actually mirror the results obtained from adequately designed animal studies carried out to high methodological standards.^28 70^

MSR and RCR scores were not influenced by the journal in which the results were published. Other researchers who have investigated the effect of journal on methodological standards and reporting quality have found a statistically significant but very small effect of whether or not the journal had endorsed the Animal Research: Reporting of In Vivo Experiments (ARRIVE) guidelines.^76 77^

In contrast to previous research, this study indicated a gradual improvement in the methodological standards and regulatory compliance reporting scores over time. However, it is difficult to say to what extent this is the result of field-specific guidelines, as there is an overall increasing trend in these scores. Our study, of course, is limited to the period and model under study, and some improvements may have occurred as a result of the informal discussion leading up to the formal workshops and guidelines (and more recently, the appearance of other transgenic models means that the study does not cover the entire field of ALS research for later years). Also, a surprisingly low number of papers (1/84 in 2009, 10/106 in 2011, 10/115 in 2013 and 14/106 in 2015) referred to the Ludolph *et al* guidelines.^2 38^ Given the slow adoption of the ARRIVE guidelines,^78^ it seems likely it may also take some time for the ALS guidelines to have a detectable effect.

While reporting of relevant parameters such as blinding and randomisation was higher in our ‘preclinical’ subsample than what has been reported in other systematic reviews,^16 31 76 78–81^ the results for the overall sample were generally comparable. Also, and similarly to what was found in these systematic reviews, justification for sample size was rarely reported.

One way of addressing the problems with study quality could be for preclinical researchers to adopt the standards of randomised controlled trials in humans,^82–85^ including trial preregistration.^86 87^ Compliance with existing guidelines would seem a more readily achievable goal; however, other self-regulatory mechanisms may be warranted to improve compliance, such as changes to the publishing requirements of biomedical journals^88–90^ or more demanding requirements by science funders, both of which are clearly on the horizon.^30 91^

## Conclusion

The ALS research community pioneered the development of field-specific guidelines, setting science community-based standards for animal research methodology and reporting.^2 38^ Whereas we found significant improvement over time, it is less clear to what extent this is linked to the guidelines, which are rarely referred to. Animal research in the field of ALS does however differ from comparable research in other reviewed fields in one aspect: the implementation of predefined endpoints in studies of advanced disease stages. This practice is important both for research quality and animal welfare and is indeed coherent with the field-specific guidelines. We propose that future guidelines should address measures to raise standards in the design, conduct and reporting of experiments, as well as to reduce the impact on animal welfare, as part of a concerted effort to make biomedical research using animals more ethically and socially acceptable and effective.

## References

[1] Miller RG , Mitchell JD , Lyon M , et al . Riluzole for amyotrophic lateral sclerosis (ALS)/motor neuron disease (MND). Amyotroph Lateral Scler Other Motor Neuron Disord 2003;4:191–206. 13129806

[2] Ludolph AC , Bendotti C , Blaugrund E , et al . Guidelines for preclinical animal research in ALS/MND: A consensus meeting. Amyotroph Lateral Scler 2010;11(1-2):38–45. 10.3109/17482960903545334 20184514

[3] Shibata N . Transgenic mouse model for familial amyotrophic lateral sclerosis with superoxide dismutase-1 mutation. Neuropathology 2001;21:82–92. 11304046 10.1046/j.1440-1789.2001.00361.x

[4] Heiman-Patterson TD , Deitch JS , Blankenhorn EP , et al . Background and gender effects on survival in the TgN(SOD1-G93A)1Gur mouse model of ALS. J Neurol Sci 2005;236(1-2):1–7. 10.1016/j.jns.2005.02.006 16024047

[5] Franco NH , Olsson IA . "How sick must your mouse be? " - An analysis of the use of animal models in Huntington’s disease research. Altern Lab Anim 2012;40:271–83. 10.1177/026119291204000506 23215663

[6] Franco NH , Correia-Neves M , Olsson IAS . Animal Welfare in Studies on Murine Tuberculosis: Assessing Progress over a 12-Year Period and the Need for Further Improvement. PLoS One 2012;7:e47723. 10.1371/journal.pone.0047723 23110093 PMC3482232

[7] Franco NH , Correia-Neves M , Olsson IAS . How “Humane” Is Your Endpoint?—Refining the Science-Driven Approach for Termination of Animal Studies of Chronic Infection. PLoS Pathog 2012;8:e1002399. 10.1371/journal.ppat.1002399 22275862 PMC3261900

[8] Solomon JA , Tarnopolsky MA , Hamadeh MJ . One Universal Common Endpoint in Mouse Models of Amyotrophic Lateral Sclerosis. PLoS One 2011;6:e20582. 10.1371/journal.pone.0020582 21687686 PMC3110799

[9] Morton DB . Humane endpoints in animal experimentation for biomedical research: ethical. legal and practical aspects. London: Royal Society of Medicine Press, 1999:5–12.

[10] Sawiak SJ , Wood NI , Williams GB , et al . Use of magnetic resonance imaging for anatomical phenotyping of the R6/2 mouse model of Huntington’s disease. Neurobiol Dis 2009;33:12–19. 10.1016/j.nbd.2008.09.017 18930823

[11] Hockly E , Woodman B , Mahal A , et al . Standardization and statistical approaches to therapeutic trials in the R6/2 mouse. Brain Res Bull 2003;61:469–79. 10.1016/S0361-9230(03)00185-0 13679245

[12] Menalled L , Brunner D . Animal models of Huntington’s disease for translation to the clinic: best practices. Mov Disord 2014;29:1375–90. 10.1002/mds.26006 25216369

[13] van der Worp HB , Howells DW , Sena ES , et al . Can Animal Models of Disease Reliably Inform Human Studies? PLoS Med 2010;7:e1000245. 10.1371/journal.pmed.1000245 20361020 PMC2846855

[14] Ioannidis JPA . Why Most Published Research Findings Are False. PLoS Med 2005;2:e124. 10.1371/journal.pmed.0020124 16060722 PMC1182327

[15] Schnabel J . Neuroscience: Standard model. Nature 2008;454:682–5. 10.1038/454682a 18685674

[16] Ioannidis JP , Greenland S , Hlatky MA , et al . Increasing value and reducing waste in research design, conduct, and analysis. Lancet 2014;383:166–75. 10.1016/S0140-6736(13)62227-8 24411645 PMC4697939

[17] Festing MFW . Randomized Block Experimental Designs Can Increase the Power and Reproducibility of Laboratory Animal Experiments. Ilar J 2014;55:472–6. 10.1093/ilar/ilu045 25541548

[18] Garner JP . The significance of meaning: why do over 90% of behavioral neuroscience results fail to translate to humans, and what can we do to fix it? Ilar J 2014;55:438–56. 10.1093/ilar/ilu047 25541546 PMC4342719

[19] Lapchak PA . Scientific Rigor Recommendations for Optimizing the Clinical Applicability of Translational Research. J Neurol Neurophysiol 2012;03. 10.4172/2155-9562.1000e111 PMC3905455 24490120

[20] Steward O , Balice-Gordon R . Rigor or Mortis: Best Practices for Preclinical Research in Neuroscience. Neuron 2014;84:572–81. 10.1016/j.neuron.2014.10.042 25442936

[21] Tsilidis KK , Panagiotou OA , Sena ES , et al . Evaluation of Excess Significance Bias in Animal Studies of Neurological Diseases. PLoS Biol 2013;11:e1001609. 10.1371/journal.pbio.1001609 23874156 PMC3712913

[22] Button KS , Ioannidis JPA , Mokrysz C , et al . Power failure: why small sample size undermines the reliability of neuroscience. Nat Rev Neurosci 2013;14:365–76. 10.1038/nrn3475 23571845

[23] Vesterinen HM , Sena ES , ffrench-Constant C , et al . Improving the translational hit of experimental treatments in multiple sclerosis. Mult Scler 2010;16:1044–55. 10.1177/1352458510379612 20685763

[24] Sena ES , van der Worp HB , Bath PMW , et al . Publication Bias in Reports of Animal Stroke Studies Leads to Major Overstatement of Efficacy. PLoS Biol 2010;8:e1000344. 10.1371/journal.pbio.1000344 20361022 PMC2846857

[25] Steward O , Popovich PG , Dietrich WD , et al . Replication and reproducibility in spinal cord injury research. Exp Neurol 2012;233:597–605. 10.1016/j.expneurol.2011.06.017 22078756

[26] Shineman DW , Basi GS , Bizon JL , et al . Accelerating drug discovery for Alzheimer’s disease: best practices for preclinical animal studies. Alzheimers Res Ther 2011;3:28. 10.1186/alzrt90 21943025 PMC3218805

[27] Kimmelman J , London AJ , Ravina B , et al . Launching invasive, first-in-human trials against Parkinson’s disease: ethical considerations. Mov Disord 2009;24:1893–901. 10.1002/mds.22712 19672990 PMC2989599

[28] Scott S , Kranz JE , Cole J , et al . Design, power, and interpretation of studies in the standard murine model of ALS. Amyotroph Lateral Scler 2008;9:4–15. 10.1080/17482960701856300 18273714

[29] Collins FS , Tabak LA . Policy: NIH plans to enhance reproducibility. Nature 2014;505:612–613. 10.1038/505612a 24482835 PMC4058759

[30] Cressey D . UK funders demand strong statistics for animal studies. Nature 2015;520:271–272. 10.1038/520271a 25877180

[31] Kilkenny C , Parsons N , Kadyszewski E , et al . Survey of the Quality of Experimental Design, Statistical Analysis and Reporting of Research Using Animals. PLoS One 2009;4:e7824. 10.1371/journal.pone.0007824 19956596 PMC2779358

[32] Crossley NA , Sena E , Goehler J , et al . Empirical evidence of bias in the design of experimental stroke studies: a metaepidemiologic approach. Stroke 2008;39:929–34. 10.1161/STROKEAHA.107.498725 18239164

[33] Hirst JA , Howick J , Aronson JK , et al . The Need for Randomization in Animal Trials: An Overview of Systematic Reviews. PLoS One 2014;9:e98856. 10.1371/journal.pone.0098856 24906117 PMC4048216

[34] van der Worp HB , Sena ES , Donnan GA , et al . Hypothermia in animal models of acute ischaemic stroke: a systematic review and meta-analysis. Brain 2007;130(12):3063–74. 10.1093/brain/awm083 17478443

[35] von Roten FC . Public perceptions of animal experimentation across Europe. Public Underst Sci 2013;22. 10.1177/0963662511428045 23885052

[36] Lund TB , Mørkbak MR , Lassen J , et al . Painful dilemmas: A study of the way the public’s assessment of animal research balances costs to animals against human benefits. Public Underst Sci 2014;23:428–44. 10.1177/0963662512451402 23825251

[37] Rollin BE . Science and Ethics: Cambridge University Press, 2006.

[38] Ludolph AC , Bendotti C , Blaugrund E , et al . Guidelines for the preclinical in vivo evaluation of pharmacological active drugs for ALS/MND: report on the 142nd ENMC international workshop. Amyotroph Lateral Scler 2007;8:217–23. 10.1080/17482960701292837 17653919

[39] Kanning KC , Kaplan A , Henderson CE . Motor Neuron Diversity in Development and Disease. Annu Rev Neurosci 2010;33:409–40. 10.1146/annurev.neuro.051508.135722 20367447

[40] Lever TE , Gorsek A , Cox KT , et al . An Animal Model of Oral Dysphagia in Amyotrophic Lateral Sclerosis. Dysphagia 2009;24:180–95. 10.1007/s00455-008-9190-z 19107538

[41] Lever TE , Simon E , Cox KT , et al . A Mouse Model of Pharyngeal Dysphagia in Amyotrophic Lateral Sclerosis. Dysphagia 2010;25:112–26. 10.1007/s00455-009-9232-1 19495873

[42] Boylan K , Yang C , Crook J , et al . Immunoreactivity of the phosphorylated axonal neurofilament H subunit (pNF-H) in blood of ALS model rodents and ALS patients: evaluation of blood pNF-H as a potential ALS biomarker. J Neurochem 2009;111:1182–91. 10.1111/j.1471-4159.2009.06386.x 19765193

[43] Kato S , Kato M , Abe Y , et al . Redox system expression in the motor neurons in amyotrophic lateral sclerosis (ALS): immunohistochemical studies on sporadic ALS, superoxide dismutase 1 (SOD1)-mutated familial ALS, and SOD1-mutated ALS animal models. Acta Neuropathol 2005;110:101–12. 10.1007/s00401-005-1019-3 15983830

[44] Gurney M , Pu H , Chiu A , et al . Motor neuron degeneration in mice that express a human Cu, Zn superoxide dismutase mutation. Science 1994;264:1772–5. 10.1126/science.8209258 8209258

[45] Bruijn LI , Becher MW , Lee MK , et al . ALS-Linked SOD1 Mutant G85R Mediates Damage to Astrocytes and Promotes Rapidly Progressive Disease with SOD1-Containing Inclusions. Neuron 1997;18:327–38. 10.1016/S0896-6273(00)80272-X 9052802

[46] Marcuzzo S , Zucca I , Mastropietro A , et al . Hind limb muscle atrophy precedes cerebral neuronal degeneration in G93A-SOD1 mouse model of amyotrophic lateral sclerosis: A longitudinal MRI study. Exp Neurol 2011;231:30–7. 10.1016/j.expneurol.2011.05.007 21620832

[47] Neymotin A , Calingasan NY , Wille E , et al . Neuroprotective effect of Nrf2/ARE activators, CDDO ethylamide and CDDO trifluoroethylamide, in a mouse model of amyotrophic lateral sclerosis. Free Radic Biol Med 2011;51:88–96. 10.1016/j.freeradbiomed.2011.03.027 21457778 PMC3109235

[48] Del Signore SJ , Amante DJ , Kim J , et al . Combined riluzole and sodium phenylbutyrate therapy in transgenic amyotrophic lateral sclerosis mice. Amyotroph Lateral Scler 2009;10:85–94. 10.1080/17482960802226148 18618304

[49] Tada S , Okuno T , Yasui T , et al . Deleterious effects of lymphocytes at the early stage of neurodegeneration in an animal model of amyotrophic lateral sclerosis. J Neuroinflammation 2011;8:19. 10.1186/1742-2094-8-19 21345177 PMC3048550

[50] Pregibon D . Goodness of Link Tests for Generalized Linear Models. Appl Stat 1980;29:15–14. 10.2307/2346405

[51] Breusch TS , Pagan AR . A Simple Test for Heteroscedasticity and Random Coefficient Variation. Econometrica 1979;47:1287–94. 10.2307/1911963

[52] Ramsey JB . Tests for Specification Errors in Classical Linear Least-Squares Regression Analysis. J Royal Stat Soc 1969;31:350–71.

[53] Franco NH , Olsson IA . Is the ethical appraisal of protocols enough to ensure best practice in animal research? Altern Lab Anim 2013;41:P5–P7. 10.1177/026119291304100117 23614553

[54] Olsson IAS , Hansen AK , Sandøe P . Animal welfare and the refinement of neuroscience research methods – a case study of Huntington’s disease models. Lab Anim 2008;42:277–83. 10.1258/la.2008.007147 18625582

[55] Lilley E , Hawkins P , Jennings M . A ’road map' toward ending severe suffering of animals used in research and testing. Altern Lab Anim 2014;42:267–72. 10.1177/026119291404200408 25290947

[56] Boillee S , Yamanaka K , Lobsiger CS , et al . Onset and Progression in Inherited ALS Determined by Motor Neurons and Microglia. Science 2006;312:1389–92. 10.1126/science.1123511 16741123

[57] Kimmelman J , Mogil JS , Dirnagl U . Distinguishing between Exploratory and Confirmatory Preclinical Research Will Improve Translation. PLoS Biol 2014;12:e1001863. 10.1371/journal.pbio.1001863 24844265 PMC4028181

[58] Benatar M . Lost in translation: Treatment trials in the SOD1 mouse and in human ALS. Neurobiol Dis 2007;26:1–13. 10.1016/j.nbd.2006.12.015 17300945

[59] Reilly J , Blackshaw AW . Euthanasia of animals used for scientific purposes: ANZCCART. 2001.

[60] Artwohl J , Brown P , Corning B , et al . ACLAM Task Force. Report of the ACLAM Task Force on Rodent Euthanasia. J Am Assoc Lab Anim Sci 2006;45:98–105. 16548095

[61] Bame M , Pentiak PA , Needleman R , et al . Effect of Sex on Lifespan, Disease Progression, and the Response to Methionine Sulfoximine in the SOD1 G93A Mouse Model for ALS. Gend Med 2012;9:524–35. 10.1016/j.genm.2012.10.014 23217569

[62] McCombe PA , Henderson RD . Effects of gender in amyotrophic lateral sclerosis. Gend Med 2010;7:557–70. 10.1016/j.genm.2010.11.010 21195356

[63] Alves CJ , de Santana LP , Santos AJDdos , AJDd S , et al . Early motor and electrophysiological changes in transgenic mouse model of amyotrophic lateral sclerosis and gender differences on clinical outcome. Brain Res 2011;1394:90–104. 10.1016/j.brainres.2011.02.060 21354109

[64] Hooijmans CR , Leenaars M , Ritskes-Hoitinga M . A gold standard publication checklist to improve the quality of animal studies, to fully integrate the Three Rs, and to make systematic reviews more feasible. Altern Lab Anim 2010;38:167–82. 10.1177/026119291003800208 20507187

[65] Banwell V , Sena ES , Macleod MR . Systematic review and stratified meta-analysis of the efficacy of interleukin-1 receptor antagonist in animal models of stroke. J Stroke Cerebrovasc Dis 2009;18:269–76. 10.1016/j.jstrokecerebrovasdis.2008.11.009 19560680

[66] Chalmers I , Glasziou P . Avoidable waste in the production and reporting of research evidence. Lancet 2009;374:86–9. 10.1016/S0140-6736(09)60329-9 19525005

[67] Festing MFW , Altman DG . Guidelines for the Design and Statistical Analysis of Experiments Using Laboratory Animals. Ilar J 2002;43:244–58. 10.1093/ilar.43.4.244 12391400

[68] Festing MF . Warning: the use of heterogeneous mice may seriously damage your research. Neurobiol Aging 1999;20:237–44. 10537033 10.1016/s0197-4580(99)00040-8

[69] Mead RJ , Bennett EJ , Kennerley AJ , et al . Optimised and Rapid Pre-clinical Screening in the SOD1G93A Transgenic Mouse Model of Amyotrophic Lateral Sclerosis (ALS). PLoS One 2011;6:e23244. 10.1371/journal.pone.0023244 21876739 PMC3158065

[70] Perrin S . Preclinical research: Make mouse studies work. Nature 2014;507:423–5. 10.1038/507423a 24678540

[71] Landis SC , Amara SG , Asadullah K , et al . A call for transparent reporting to optimize the predictive value of preclinical research. Nature 2012;490:187–91. 10.1038/nature11556 23060188 PMC3511845

[72] Bara M , Joffe AR . The methodological quality of animal research in critical care: the public face of science. Ann Intensive Care 2014;4:1–9. 10.1186/s13613-014-0026-8 25114829 PMC4126494

[73] Howells DW , Macleod MR . Evidence-based Translational Medicine. Stroke 2013;44:1466–71. 10.1161/STROKEAHA.113.000469 23559263

[74] Bebarta V , Luyten D , Heard K . Emergency medicine animal research: does use of randomization and blinding affect the results? Acad Emerg Med 2003;10:684–7. 10.1197/aemj.10.6.684 12782533

[75] Macleod MR , van der Worp HB , Sena ES , et al . Evidence for the Efficacy of NXY-059 in Experimental Focal Cerebral Ischaemia Is Confounded by Study Quality. Stroke 2008;39:2824–9. 10.1161/STROKEAHA.108.515957 18635842

[76] Avey MT , Moher D , Sullivan KJ , et al . The Devil Is in the Details: Incomplete Reporting in Preclinical Animal Research. PLoS One 2016;11:e0166733. 10.1371/journal.pone.0166733 27855228 PMC5113978

[77] Vogt L , Reichlin TS , Nathues C , et al . Authorization of Animal Experiments Is Based on Confidence Rather than Evidence of Scientific Rigor. PLoS Biol 2016;14:e2000598. 10.1371/journal.pbio.2000598 27911892 PMC5135031

[78] Baker D , Lidster K , Sottomayor A , et al . Two Years Later: Journals Are Not Yet Enforcing the ARRIVE Guidelines on Reporting Standards for Pre-Clinical Animal Studies. PLoS Biol 2014;12:e1001756. 10.1371/journal.pbio.1001756 24409096 PMC3883646

[79] Gulin JEN , Rocco DM , García-Bournissen F . Quality of Reporting and Adherence to ARRIVE Guidelines in Animal Studies for Chagas Disease Preclinical Drug Research: A Systematic Review. PLoS Negl Trop Dis 2015;9:e0004194. 10.1371/journal.pntd.0004194 26587586 PMC4654562

[80] Macleod MR , Lawson McLean A , Kyriakopoulou A , et al . Risk of Bias in Reports of In Vivo Research: A Focus for Improvement. PLoS Biol 2015;13:e1002273. 10.1371/journal.pbio.1002273 26460723 PMC4603955

[81] Ting KHJ , Hill CL , Whittle SL . Quality of reporting of interventional animal studies in rheumatology: a systematic review using the ARRIVE guidelines. Int J Rheum Dis 2015;18:488–94. 10.1111/1756-185X.12699 26082348

[82] Muhlhausler BS , Bloomfield FH , Gillman MW . Whole Animal Experiments Should Be More Like Human Randomized Controlled Trials. PLoS Biol 2013;11:e1001481. 10.1371/journal.pbio.1001481 23424284 PMC3570551

[83] McGonigle P , Ruggeri B . Animal models of human disease: Challenges in enabling translation. Biochem Pharmacol 2014;87:162–71. 10.1016/j.bcp.2013.08.006 23954708

[84] Hackam DG . Translating animal research into clinical benefit. BMJ 2007;334:163–4. 10.1136/bmj.39104.362951.80 17255568 PMC1782020

[85] de Vries RBM , Wever KE , Avey MT , et al . The Usefulness of Systematic Reviews of Animal Experiments for the Design of Preclinical and Clinical Studies. Ilar J 2014;55:427–37. 10.1093/ilar/ilu043 25541545 PMC4276599

[86] Jansen of Lorkeers SJ , Doevendans PA , Chamuleau SA . All preclinical trials should be registered in advance in an online registry. Eur J Clin Invest 2014;44:891–2. 10.1111/eci.12299 25041644

[87] Dal-Re R , Ioannidis JP , Bracken MB , et al . Making Prospective Registration of Observational Research a Reality. Sci Transl Med 2014;6:224cm1. 10.1126/scitranslmed.3007513 24553383

[88] Rollin BE . University of Miami / Georgia Tech. Animal Research, Animal Welfare, and the Three R’s. J Philos Sci Law 2010;10:1–11. 10.5840/jpsl20101022

[89] Osborne NJ , Phillips BJ , Westwood K . Journal editorial policies as a driver for change - animal welfare and the 3R. New Paradigms. Proceedings of the Eleventh FELASA symposium and the 40th Scand-LAS Symposium. Helsinki, 2010:18–23. Laboratory Animal Science.

[90] Martins A , Franco N . A Critical Look at Biomedical Journals’ Policies on Animal Research by Use of a Novel Tool: The EXEMPLAR Scale. Animals 2015;5:315–31. 10.3390/ani5020315 26479237 PMC4494415

[91] Editorial. Checklists work to improve science. Nature 2018;556:273–4. 10.1038/d41586-018-04590-7 30967653

